# Cytochrome P450 3A4 suppression by epimedium and active compound kaempferol leads to synergistic anti-inflammatory effect with corticosteroid

**DOI:** 10.3389/fphar.2022.1042756

**Published:** 2023-01-30

**Authors:** Ke Li, Xiu-Hua Yu, Anish R. Maskey, Ibrahim Musa, Zhen-Zheng Wang, Victor Garcia, Austin Guo, Nan Yang, Kamal Srivastava, David Dunkin, Jun-Xiong Li, Longgang Guo, Yung-Chi Cheng, Haoliang Yuan, Raj Tiwari, Xiu-Min Li

**Affiliations:** ^1^ Guangdong Hospital of Integrated Traditional Chinese and Western Medicine, Guangzhou University of Chinese Medicine, Foshan, China; ^2^ Department of Pathology, Microbiology and Immunology, New York Medical College, Valhalla, NY, United States; ^3^ Central Laboratory, Affiliated Hospital, Changchun University of Chinese Medicine, Changchun, China; ^4^ Academy of Chinese Medical Science, Henan University of Chinese Medicine, Zhengzhou, China; ^5^ Department of Pharmacology, New York Medical College, Valhalla, NY, United States; ^6^ General Nutraceutical Technology, Elmsford, NY, United States; ^7^ Department of Pediatrics, Division of Gastroenterology, Icahn School of Medicine at Mount Sinai, New York, NY, United States; ^8^ Guangzhou ImVin Pharmaceutical Co., Ltd., Guangzhou, China; ^9^ Department of Pharmacology, School of Medicine, Yale University, New Haven, China; ^10^ Jiangsu Key Laboratory of Drug Discovery for Metabolic Disease and State Key Laboratory of Natural Medicines, China Pharmaceutical University, Nanjing, China; ^11^ Department of Otolaryngology, Westchester Medical Center New York Medical College, Valhalla, NY, United States

**Keywords:** epimedium, CYP3A4, drug-drug interaction (DDI), anti-inflammation, kaempferol

## Abstract

**Introduction:** Cytochrome P450 (CYP) 3A4 is a major drug metabolizing enzyme for corticosteroids (CS). Epimedium has been used for asthma and variety of inflammatory conditions with or without CS. It is unknown whether epimedium has an effect on CYP 3A4 and how it interacts with CS. We sought to determine the effects of epimedium on CYP3A4 and whether it affects the anti-inflammatory function of CS and identify the active compound responsible for this effect.

**Methods:** The effect of epimedium on CYP3A4 activity was evaluated using the Vivid CYP high-throughput screening kit. CYP3A4 mRNA expression was determined in human hepatocyte carcinoma (HepG2) cells with or without epimedium, dexamethasone, rifampin, and ketoconazole. TNF-α levels were determined following co-culture of epimedium with dexamethasone in a murine macrophage cell line (Raw 264.7). Active compound (s) derived from epimedium were tested on IL-8 and TNF-α production with or without corticosteroid, on CYP3A4 function and binding affinity.

**Results:** Epimedium inhibited CYP3A4 activity in a dose-dependent manner. Dexamethasone enhanced the expression of CYP3A4 mRNA, while epimedium inhibited the expression of CYP3A4 mRNA and further suppressed dexamethasone enhancement of CYP3A4 mRNA expression in HepG2 cells (*p* < 0.05). Epimedium and dexamethasone synergistically suppressed TNF-α production by RAW cells (*p* < 0.001). Eleven epimedium compounds were screened by TCMSP. Among the compounds identified and tested only kaempferol significantly inhibited IL-8 production in a dose dependent manner without any cell cytotoxicity (*p* < 0.01). Kaempferol in combination with dexamethasone showed complete elimination of TNF-α production (*p* < 0.001). Furthermore, kaempferol showed a dose dependent inhibition of CYP3A4 activity. Computer docking analysis showed that kaempferol significantly inhibited the catalytic activity of CYP3A4 with a binding affinity of −44.73kJ/mol.

**Discussion:** Inhibition of CYP3A4 function by epimedium and its active compound kaempferol leads to enhancement of CS anti-inflammatory effect.

## 1 Introduction

The impact of a drug on the efficacy, toxicity, or metabolism of another commonly referred to as drug-drug interaction, has presented a challenge in managing patient’s healthcare around the world (Tornio et al., 2019). Better understanding of the combination of Chinese herbal medicines and synthetic drugs could improve the efficacy of clinical medications and reduce the occurrence of adverse reactions. Drug-metabolizing enzymes are the main component of human detoxification and bioavailability systems, with several hundred types having been identified to date (Preissner et al., 2013). Among these, cytochrome P450(CYP) proteins have been shown to be involved in most drug metabolism reactions, with CYP3A4 being the most important in the human liver where it facilitates 30%–40% of drug metabolization (Shimada et al., 1994; Paine et al., 2006; Zanger and Schwab, 2013). Some drugs, such as dexamethasone (Dex), metabolized by CYP3A4, are known to enhance CYP3A4 activity and accelerate drug metabolism, which may reduce the efficacy of drugs and lead to disappointing anti-inflammatory effects (Pascussi et al., 2001).

Corticosteroids (CS) are the most widely used benchmark anti-inflammatory medication and the first line of therapy for a variety of chronic inflammatory autoimmune and allergic disorders such as asthma, lupus, Crohn’s disease, and atopic dermatitis (Chong and Fonacier, 2016; Adcock and Mumby, 2017). While extremely efficacious, prolonged use of CS to control chronic symptoms and the necessity of using it in long-term or at high doses for the management of difficult-to-control diseases is problematic due to the high risk of serious adverse effects. The side-effects of high-dose CS use include global immune suppression, disruption of the hypothalamic pituitary adrenal (HPA) axis, loss of bone density, increased water retention, dyslipidemia, and dermal thinning (Yasir et al., 2022). Chuan Ke Zhi (CKZ) is a China Food and Drug Administration approved medication (Imvin Pharmaceutical LTD., Guangzhou, China) comprised of epimedium and Morinda officinalis (Ba Ji Tian) (Zhao et al., 2013). *Epimedium sagittatum* is a key herbal constituent from CKZ that has been approved by the China FDA for treating osteoporosis, sexual dysfunction, hypertension, and common inflammatory diseases, such as chronic obstructive pulmonary disease and asthma particularly steroid-resistant asthma (Zhao et al., 2013; Xu et al., 2016). Yet, it is unknown whether epimedium can influence CYP3A4 or how it interacts with CS. We tested 141 TCM natural products using Vivid® CYP high-throughput screening kit (Invitrogen Corporation; Carlsbad, CA). Epimedium is one of the natural products that showed over 90% inhibition of CYP3A4 (Yu et al. manuscript in preparation). Consequently, an increased understanding of these interactions will ensure appropriate prescription medicines for long-term treatments and appropriate dosing, especially for the use of CS.

The objective of this study was to determine the effects of epimedium on CYP3A4 function and mRNA expression. Moreover, we sought to determine the relationship between epimedium and dexamethasone with regards to changes in CYP3A4 mRNA expression and its anti-inflammatory effects. To this end, we accessed epimedium’s effect on CYP3A4 activity, as well as its effect on CYP3A4 mRNA expression and on the production of the inflammatory cytokine, TNF-α with or without dexamethasone. We further identified bioavailable compounds based on the traditional Chinese medicine system pharmacology database and analysis platform (TCMSP) and tested their anti-IL-8 and anti- TNF-α effects in a human liver cell line and mouse macrophage cell line with or without dexamethasone. The most effective compound, kaempferol was further subjected to assessment of CYP3A4 function by Vivid assay and molecular binding to the CYP3A4 protein structure by computational docking. We, for the first time, demonstrated that epimedium and its active compound kaempferol exhibit an inhibitory effect on CYP3A4, the key CS drug metabolizing enzyme, leading to a synergistic anti-inflammatory effect with dexamethasone.

## 2 Materials and methods

### 2.1 Sources of *epimedium sagittatum* extract, active compounds, solvents, and cell lines

According to FDA, the impurity for extract from the natural source means “elemental impurities” (FDA. Botanical, 2016). The powdered water extract of *epimedium sagittatum* (Yin Yang Huo) used in our *in vitro* experiments was made by ImVin Pharmaceutical Co., Ltd. (Guangzhou, China). Epimedium extract was produced in a good manufacturing practice (GMP) facility (Epimedium processing method is shown in Supplementary Method S1). The quality was monitored by high-performance liquid chromatography (HPLC) and thin layer chromatography (Supplementary Figure S1; Supplementary Figure S2), and the heavy metal content, pesticide residual and microbial levels all meet FDA standards. Epimedium extract was dissolved in corning phosphate buffered saline (PBS, Fisher scientific, MA, United States) for cell culture experiments. The pure compounds, Icariin, anhydroicaritin, quercetin, and kaempferol (Chengdu Herbpuritfy Co., Sichuan, China) with a purity >98% were generous gifts from United States Time Technology Inc. (Elmsford, NY). Compounds were dissolved in dimethyl sulfoxide (DMSO, Fisher Scientific) for *in vitro* cell culture. The concentration of DMSO used in the final culture concentration is less than .1%. This method has been widely used in previous publications including ours (Lopez-Exposito et al., 2011; Yang et al., 2014; Srivastava et al., 2020). HepG2 cells and RAW264.7 cells were purchased from American Tissue Culture Company (Manassas, Virginia).

### 2.2 Measurement of CYP3A4 activity by Vivid CYP450 assay kits

Vivid CYP450 assay kits were purchased from Thermo Fisher Scientific, Waltham, Massachusetts. This kit has been widely used to measure CYP3A4 activity. The stock solution of epimedium (100 mg/mL) was prepared in methanol and diluted to final concentrations of epimedium (500 μg/mL) with P450 reaction buffer. The positive control compound was ketoconazole (final concentration was 10 μM). In each well, 40 µL of epimedium, solvent or a positive inhibitor control were incubated with 50 µL of pre-mixture at room temperature (25°C) for 20 min. The reaction was initiated by the addition of 10 µL/well of a mixture of substrate and NADP (+) with the appropriate concentration of the vivid substrate. The plate was read immediately for fluorescence changes every 2 min for 60 min using a Spectra Max iD5 Multi-Mode Micro plate Reader (Molecular Devices; San Jose, CA, United States) with appropriate excitation and emission wavelengths for 490 nm and 520 nm. Based on the Vivid CYP450 kits user guide, we used the below formula to calculate percent inhibition.

$$\%Inhibition=\left(1-\frac{X-B}{A-B}\right)\times 100\%$$

Where X is the rate observed in the presence of test compound, A is the rate observed in the presence of inhibitor (solvent control or no inhibitor control, as appropriate), and B the rate observed in the presence of the positive inhibition control, ketoconazole. In aspirate experiment, the active compound (kaempferol at the concentrations of 5, 10, 20, and 40 μg/mL) from epimedium was measured for its effect on CYP3A4 activity by Vivid CYP450 assay kits following the same method as per manufacturer’s instruction, (Themo Fisher Scientific, Waltham, Massachusetts) and previous publication (Li et al., 2013). All experiments were replicated at least three times.

### 2.3 HepG2 cell culture and quantitative reverse transcription polymerase chain reaction (qRT-PCR) assay to determine CYP3A4 mRNA expression

HepG2 cells, an immortalized cell line consisting of human liver carcinoma cells, derived from the liver tissues of a 15-year-old Caucasian male who had a well-differentiated hepatocellular carcinoma, were cultured in Dulbecco’s Modified Eagle’s Medium (DMEM) containing 10% Fetal Bovine Serum (FBS), 100 U/m L Penicillin and 100 μg/mL Streptomycin. Cell cultures were maintained in a humidified 37°C, 5% CO_2_ incubator. In-order to measure CYP3A4 expression, HepG2 cells (5 × 10^5^ cells/mL) were added in a 6-well tissue culture plate. After 24 h of incubation at 37°C in 5% CO_2_, the cells were treated with vehicle (0.1%DMSO), rifampin(10 µM), ketoconazole(10 µM), dexamethasone(10 µM), epimedium (125 μg/mL), and dexamethasone (10 µM) in combination with epimedium (125 μg/mL) in 1 mL of medium for 48 h. RNA was extracted using AllPrep DNA/RNA Mini Kit (QIAGEN company) according to the manufacturer’s recommended protocol. Total RNA was subjected to reverse transcription using RevertAid RT Kit (Themofisher Scientific) and the following primer pairs: CYP3A4, forward 5′-TGG​AAG​AGA​TTA​CGA​TCA​TTG​CT-3′ and reverse 5′-AGT​CGA​TGT​TCA​CTC​CAA​ATG​AT-3′ was used to perform q-RT PCR using SYBR® Green PCR Master Mix (Themofisher Scientific). All experiments were replicated at least three times. A similar experiment was performed using kaempferol. HepG2 cells (5 × 10^5^ cells/mL) were cultured with vehicle (0.1%DMSO), dexamethasone(0.1 µM), dexamethasone(0.1 µM), kaempferol (40 μg/mL), dexamethasone (0.1 µM) combined with kaempferol (40 μg/mL), and dexamethasone (1 µM) combined with kaempferol (40 μg/mL) for 48 h after which RNA was extracted and expression of CYP3A4 was determined using the same qRT-PCR kit and primers as described above. All experiments were replicated at least three times.

### 2.4 Western blotting

In order to measure CYP3A4 protein expression, HepG2 cells, were cultured in Dulbecco’s Modified Eagle’s Medium (DMEM) containing 10% Fetal Bovine Serum (FBS), 100 U/m L Penicillin and 100 μg/mL Streptomycin. Cell cultures were maintained in a humidified 37°C, 5% CO_2_ incubator. HepG2 cells at 3 × 10^6^ cells/mL were added in a 6-well tissue culture plate. After 24 h of incubation at 37°C in 5% CO_2_, the cells were treated with vehicle (0.1%DMSO), epimedium(125 μg/mL) for 48 h. After the culture time point the cells were harvested, centrifuged at 3,000 rpm for 5 min and the supernatant discarded. The cell suspension was washed with PBS and centrifuged at 3,000 rpm for 5 min. Then the cell lysis was done for protein extraction using 100–200 µL of RIPA lysis buffer, with intermittent vigorous vortexing and incubating on ice for about 1 h. After completion of lysis, the tubes were spined in centrifuge at 14,000 rpm for 20 min at 4°C. The supernatant was collected and transferred to another tube to measure the protein concentration. Electrophoresis was done at 90 V for 15 min followed by 100–110 V for the remaining time and transferred to a PVDF membrane. The membrane was incubated with primary antibodies of the CYP3A4, β-actin overnight. The next day, the membrane was washed with PBST and incubated with secondary antibody for 2 h and after the membrane was washed with PBST 4 times before adding the chemiluminescence substrate and imaging. The primary antibodies of CYP3A4 and β-actin were ordered from Cell Signaling (Danvers, MA, United States) and were used at a 1:1,000 dilution, the secondary antibody was used at a 1:10,000 dilution.

### 2.5 Cell proliferation and viability

HepG2 cells at the concentration of 5 × 10^3^cells/well/100 μL were added in a 96-well tissue culture plate to adhere and incubated at 37°C with 5% CO_2_ for 24 h. Cells were treated with different concentrations of epimedium (500 μg/mL, 250 μg/mL, 125 μg/mL, 62.5 μg/mL, 31.25 μg/mL) for 48 h 20µL of MTT 3-(4,5-Dimethylthiazol-2-yl)-2,5-Diphenyltetrazolium Bromide, 5ug/mL, Thermofisehr, NJ, United States) was added to each well and incubated for 4 h after which the supernatants were discarded and replaced with 150 µL of DMSO. The plate was placed in an orbital shaker at room temperature for 10 mins and absorbance was read at 595 nm using Vmax Kinetic ELISA Micro plate Reader. The percentage of cell proliferation was calculated by comparison with the vehicle (0.1% DMSO) which was set at 100%.

In a separate experiment, HepG2 cells (6.5 × 10^3^ cells/well/100 μL) were added in a 96-well tissue culture plate to adhere for 24 h (37°C with 5% CO_2_). Then cells were incubated with different treatments (dexamethasone at 0.1 µM, 1 μM, kaempferol at 40 μg/mL, kaempferol at 40 μg/mL combined with 0.1 μM and 1 μM dexamethasone) for 48 h 10 µL of CCK8 was added in each well. After 4 h of incubation at 37°C, absorbance was read at 450 nm using a Micro plate Reader. All experiments were replicated at least three times.

### 2.6 TNF- α measurement

RAW264.7 cells are murine macrophage cells that produce TNF- α and have been used to measure changes in TNF- α production after treatments (Liu et al., 2015). RAW264.7 growth medium is composed of DMEM with 10% FBS and 1% penicillin/streptomycin and was changed every 3–4 days or at 75% confluency. Raw 264.7 cells were diluted to a concentration of 2 × 10^5^ cells/mL and plated with 250 μL in 48-well plates. The cells were incubated at 37°C with 5% CO2 for 1 h to allow their attachment to the bottom of the wells. Treatments of epimedium at 125 μg/mL and prescribed concentrations of 10^−6^ M or 10^−7^ M dexamethasone (Sigma Aldrich, St. Louis, MO), were added in volumes of 200 μL. .5 μg/mL of LPS (Escherichia coli0111: B4, Sigma Aldrich, St. Louis, MO) were also added for stimulation. The cells were then incubated at 37°C with 5% CO2 for 24 h and the supernatants were subsequently collected for TNF- α quantification using a mouse TNF- α ELISA kit (BD Biosciences). The dilution of TNF-α measurement was 1:30. All experiments were replicated at least three times with duplicate ELISA measurements of each sample.

### 2.7 Screening for epimedium active compounds using the TCMSP data base

TCMSP is a unique systems pharmacology platform of Chinese herbal medicines that captures the relationships between drugs, targets, and diseases. Oral bioavailability (OB) is one of the most crucial pharmacokinetic properties frequently evaluated in early drug screening which represents the percentage of an orally administered dose of a given compound that delivers to the systemic circulation to produce a pharmacological effect in the organism. High OB is a pivotal factor in determining bioactive molecules as a potential therapeutic agent. Drug-likeness (DL) is a qualitative means of analysis used in drug design for determining the drug ability of a given molecule, which is an important consideration in guiding the design and selection of compounds during the early stages of drug discovery and development.

### 2.8 IL-8 measurement

HepG2 cells were diluted to a concentration of $5\times 10^{5}$ cells/mL and plated with 1 m L in 12-well plates. The cells were incubated at 37°C with 5% CO_2_ for 1 h to attach to the bottom of the wells. Kaempferol at 40 μ g/mL and prescribed concentrations of 10^–6^ M or 10^–7^ M dexamethasone (Sigma Aldrich, St. Louis, MO), were added in volumes of 500 μ L. After pre-treatment for 48 h, 5 ng/mL of IL-1β was added to pre-treatment groups for stimulation. The cells were incubated at 37°C with 5% CO_2_ for 6 h and the supernatants were subsequently collected for IL-8 quantification using a human IL-8 ELISA kit (BD Biosciences). The dilution of IL-8 measurement was 1:1. All experiments were replicated at least three times with duplicate ELISA measurements of each sample.

### 2.9 Computer docking for kaempferol and CYP3A4

The crystal structure of P450 3A4 (PDB ID: 2V0M) was obtained from the protein data bank (http://www.rcsb.org/). CDOCKER implemented in Discovery Studio 3.0 has been used for molecular docking. The force field has been applied to the crystal structure. All the other molecules, including water, were removed. The crystal ligand was used as the reference to define the binding site with the coordinate of 18.9801, 8.89066, 61.9966. The size of site sphere was defined with a radius of 12 Å. All the compounds were prepared using the Prepare Ligands protocol. Finally, the docking poses for each compound were outputted based on the docking score.

The crystal structure of P450 3A4 (PDB ID: 2V0M) was obtained from the protein data bank (http://www.rcsb.org/). CDOCKER implemented in Discovery Studio 3.0 has been used for molecular docking. The force field has been applied to the crystal structure. All the other molecules, including water, were removed. The crystal ligand was used as the reference to define the binding site with the coordinate of 18.9801, 8.89066, 61.9966. The size of site sphere was defined with a radius of 12 Å. The molecular structures of Epimedium components were obtained from NCBI and their SDF file format were downloaded from TCMSP. All the compounds were prepared using the Prepare Ligands protocol. Finally, the docking poses for each compound were determined based on the docking score.

## 3 Results

### 3.1 Epimedium’s dose-dependent inhibition of CYP3A4 activity

**FIGURE 1:**
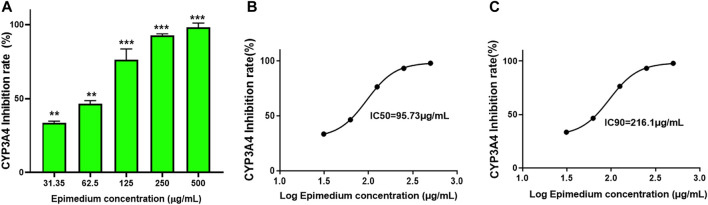
Epimedium inhibition of CYP3A4 activity: The inhibition of CYP3A4 was measured by commercially available Vivid assay kit as per manufacturers instruction. Epimedium showed dose-dependent suppression of CYP3A4 enzyme activity **(A)**. The IC_50_ and IC_90_ were calculated to be 95.73 pg/mL and 216.1 pg/mL respectively **(B,C)**. Data represents triplicate experiments and expressed as mean ± SD. np < 0.01; ***p < 0.001 *vs.* control.

To determine if epimedium inhibits CYP3A4 activity, a Vivid CYP450 kit was used to measure the effect of different concentrations of epimedium. Results showed that epimedium suppressed CYP3A4 activity in a dose-dependent manner. The inhibition rate was 33.5%, 45.5%, 76.3%, 93.2%, and 98% at an incremental concentration of 31.25, 62.5, 125, 250, and 500 μg/mL, respectively (Figure 1A). According to the epimedium inhibition of CYP3A4, the calculated IC50 and IC90 was 95.74 μg/mL and 216.1 ug/mL, respectively (Figures 1B, C). These results demonstrate that epimedium exhibits a potent inhibitory effect on CYP3A4 activity.

### 3.2 Epimedium suppressed CYP3A4 mRNA expression and reduced dexamethasone enhancement of CYP3A4 mRNA expression without cytotoxicity in a human liver carcinoma cell line (HepG2)

**FIGURE 2:**
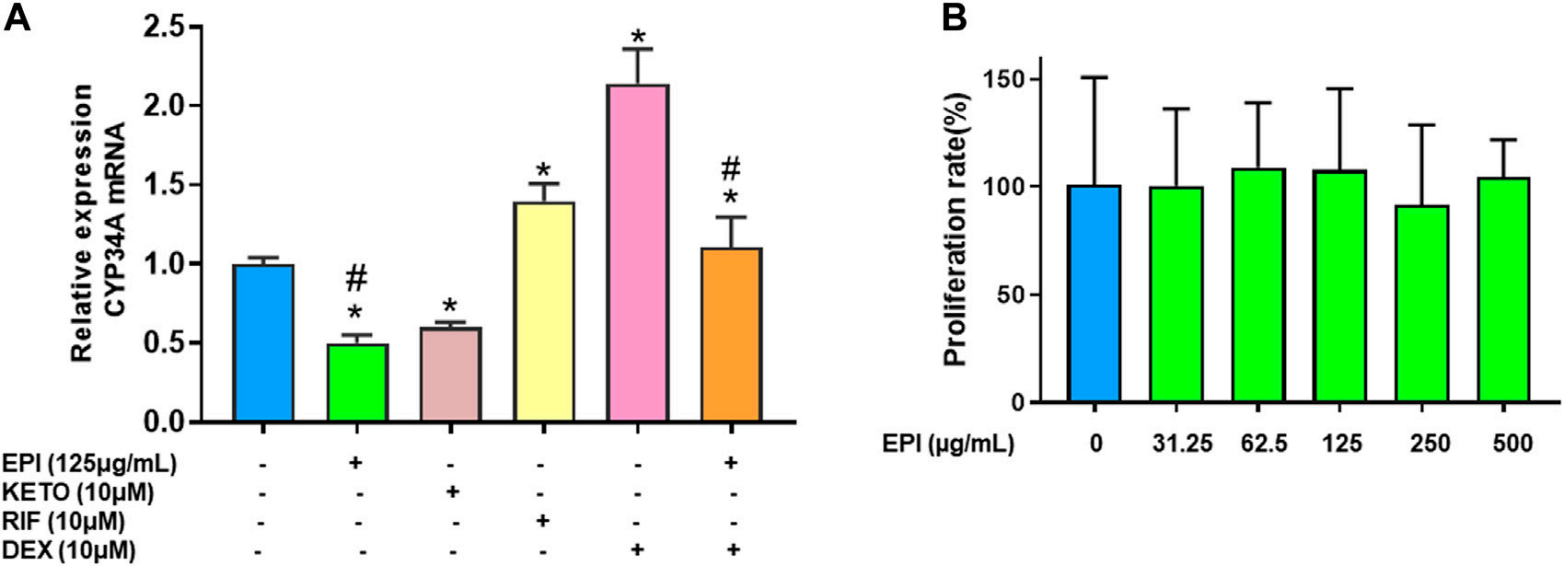
Epimedium suppressed CYP3A4 mRNA expression in human liver cell lines: HepG2 cells were cultured with Epimedium (EPI) and Dexamethasone (DEX) either alone or in combination. Rifampin (RI F)was used as a CYP3A4 enhancer whereas Ketoconazole (KETO) as a CYP3A4 inhibitor. The expression levels were determined by qRT-PCR. EPI alone inhibited CYP3A4 mRNA expression comparable to KETO whereas DEX enhanced CYP3A4 mRNA levels comparable to RIF. Coculture EPI and DEX remarkably reduced DEX enhancement of CYP3A4 mRNA levels **(A)**. Cell proliferation assay measured using MTT assay showed no different in proliferation between groups **(B)**. Data represents triplicate experiments and expressed as mean ± SD. *p < .05 *vs.* control (0.1%DMS0), #*p* < 0.05 *vs*. DEX.

To determine if epimedium suppresses CYP3A4 mRNA expression, we conducted quantitative PCR in HepG2 cells (liver carcinoma cell line). Epimedium, like ketoconazole, significantly inhibited CYP3A4 gene expression by 50% and 40% respectively (Figure 2A, *p* < 0.05 *vs.* DMSO control). In contrast, rifampin, and dexamethasone alone significantly increased CYP3A4 mRNA level by 39% and 114% respectively (*p* < 0.05 *vs*. DMSO control). However, when epimedium was used in combination with dexamethasone, we saw a 50% suppression of the CYP3A mRNA expression in HepG2 cells (*p* < 0.05). We next conducted an MTT assay to examine whether epimedium inhibition of CYP3A4 expression is linked to a cytotoxic effect. MTT is a well accepted colorimetric assay to determine cell proliferation, viability, and cytotoxicity. We observed that there was no significant difference in the rate of proliferation/viability in HepG2 cells at 31.25, 62.5, 125, 250 and 500 μg/mL concentration (Figure 2B). Taken together, this data demonstrates that epimedium inhibits CYP3A4 mRNA expression, and counter-regulates the dexamethasone paradoxical enhancement of CYP3A4 in a non-cytotoxic manner.

### 3.3 Epimedium suppressed CYP3A4 protein expression in a human liver carcinoma cell line (HepG2)

**FIGURE 3:**
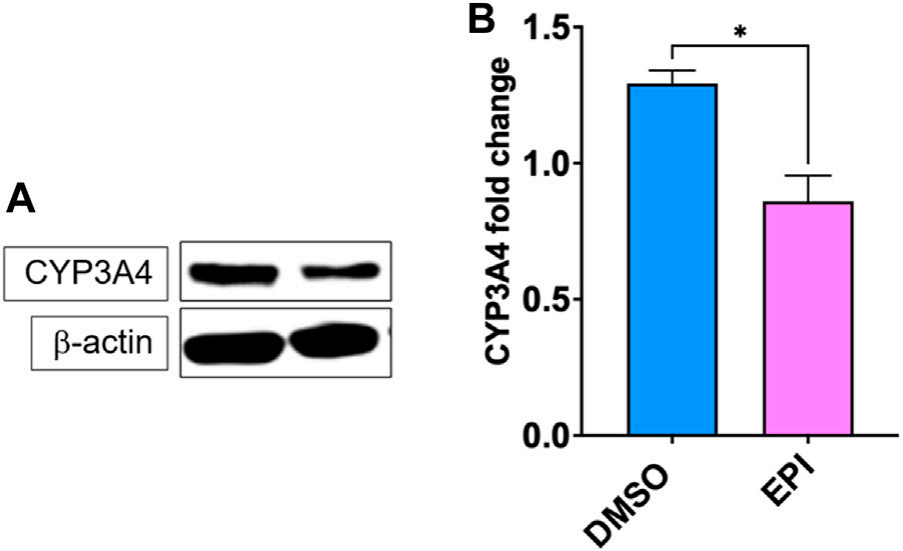
Epimedium inhibits CYP3A4 protein expression. HepG2 cells were cultured with Epimedium for 48 h and protein expression was determined using western blotting. **(A)**. Western Bands **(B)**. CYP3A4 protein expression. Data represents triplicate experiments and expressed as mean ± SD. *p < .05 *vs*. control (0.1%DMS0).

Epimedium suppressed CYP3A4 mRNA expression, therefore we used western blotting to measure the protein expression of CYP3A4. Our results showed that epimedium significantly inhibited CYP3A4 protein expression (Figure 3, *p* < 0.05).

### 3.4 Epimedium and dexamethasone synergistically suppressed TNF-α production by murine macrophage cell line (Raw 264.7)

**FIGURE 4:**
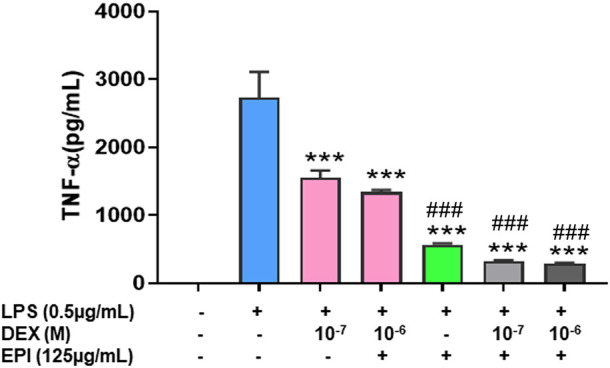
Epimedium and dexamethasonesynergistically suppressed TNF-α production: RAW cells were cultured with Epimedium either alone or in combination with Dexamethasone for 24 h. The cells were stimulated with LPS (0.5 pg/mL). The supernatant were harvested and TNF-a levels were measured by ELISA. **(A)** Dexamethasone (10^−6^ M, 10^−7^ M) and Epimedium (125 pg/m1) significantly reduced TNF-α levels. When Epimedium was added to dexamethasone, the TNF-α levels were suppressed by 88%. Data represents triplicate experiments and expressed as mean ± SD. ***p < 0.001 *vs*. control (0.1%DMS0),"#*p* < 0.001 *vs*. Dex (10–7 M).

TNF-α is a master pro-inflammatory cytokine and plays a major pathological role in corticosteroid resistant asthma, inflammatory bowel disease, psoriasis, rheumatoid arthritis and COVID-19 cytokine storm among others (Russell et al., 2020). Since we observed that dexamethasone upregulated CYP3A4 mRNA expression and that may be counterproductive to its anti-inflammatory effect, and epimedium reduced this unwanted effect of dexamethasone on CYP3A4 mRNA, we hypothesize that epimedium may enhance the anti-inflammatory effect of dexamethasone. To test this possibility, we used murine macrophage cell line, Raw 264.7 cells. Our results showed that dexamethasone at 10^-−7^ M only inhibited TNF-α by 20.4%, compared with that in the untreated cells. Co-treatment of dexamethasone (10^–6^ M) and epimedium (125 μg/mL) significantly decreased TNF-α level (stimulated by LPS (500 ng/mL)) by 63.2% and 78.3% respectively (Figure 4, *p* < 0.01). Interestingly, when epimedium (125 μg/mL) was added to dexamethasone at either 10^−7^ M or 10^–6^ M, we observed that the TNF-α levels were significantly lower than either dexamethasone or epimedium alone (*p* < 0.001). These data clearly demonstrates that epimedium provided synergistic effect with dexamethasone on inhibition of TNF-α production.

### 3.5 Screening bioavailable active compounds from epimedium *via* TCMSP database

We used the TCMSP database to filter potential bioavailable active components in epimedium (Ru et al., 2014). The screening criteria were oral bioavailability (OB) > 40% and drug like (DL) index>0.18, as previously described (Ru et al., 2014; Wang et al., 2021a; Wang et al., 2021b). Eleven potential compounds were identified. The molecular names of these compounds are magnograndiolide, olivil, Yinyanghuo A, 1,2-diguaiacylpropane-1,3-diol, Yinyanghuo E, quercetin, Yinyanghuo C, anhydroicaritin, linoleyl acetate, kaempferol and icariin. The molecular weight of all potential epimedium compounds identified fall between 250–500 g/mol except for Icariin which was around 676.73 g/mol (Table 1).

**TABLE 1:** Active compounds from Epimedium.

Index	Molecule name	Molecule wt. (g/Mol)	OB(%)>40	DL > .18
1	Magnograndiolide	266.37	63.71	.19
2	olivil	376.44	62.23	.41
3	Yinyanghuo A	420.49	56.96	.77
4	1,2-diguaiacylpropane-1,3-diol	320.37	52.31	.22
5	Yinyanghuo E	352.36	51.63	.55
6	quercetin	302.25	46.43	.28
7	Yinyanghuo C	336.36	45.67	.5
8	Anhydroicaritin	368.41	45.41	.44
9	Linoleyl acetate	308.56	42.1	0.2
10	Kaempferol	286.25	41.88	.24
11	Icariin	676.73	41.58	.61

Screening active compounds from Epimedium using TCMSP, database: The screening criteria were OB>40% and DL > 0.18. We found 11 active compounds of Epimedium. All the molecule weights were between 250 and 676.73 (g/Mol). TCMSP.

### 3.6 Effect of 4 epimedium candidate compounds on IL-8 production by HepG2 cells

**FIGURE 5:**
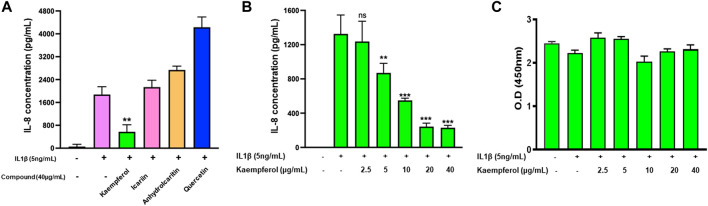
Effect of four epimedium candidate compounds on IL-8 production: HepG2 cells were cultured in presence of four main Epimedium candidate compound for 48 h. After which cells were stimulated with IL-1 β (5 ng/m1). The supernatant were harvest and IL-8 levels were measured by ELISA. **(A)** Out of four main candidates, Kaempferol significantly reduced IL-8 levels. **(B)** Kaempferol showed a dose dependent inhibition of IL-8 production by stimulated HepG2 cells. **(C)** No cell cytotoxicity was observed with Kaempferol at different doses. Data represents triplicate experiments and expressed as mean ± SD. "*p* < 0.01; *p* < 0.001; *vs*. control (0.1%DMSO). 0.D; optical density.

Next, we tested epimedium derived compounds from the TCMSP screening results and focused on four commercially available compoundsicariin, anhydroicaritin, quercetin, and kaempferol. We tested these compounds at a concentration of 40 μg/mL against IL-1β-stimulated HepG2 cells and determined levels of the of the inflammatory cytokine IL-8. We found that quercetin enhanced IL-8 levels, opposite to the parent extract (Figure 5A). Icariin and anhydroicaritin did not reduce IL-8 production at the tested doses. Only kaempferol showed significant inhibition of IL-8 production (*p* < 0.01). We further showed a dose dependent reduction of IL-8 production by kaempferol (Figure 5B, *p* < 0.01–0.001) with no cytotoxicity (Figure 5C).

### 3.7 Kaempferol and dexamethasone combined effects onTNF-α production in the murine macrophage cell line (Raw264.7)

**FIGURE 6:**
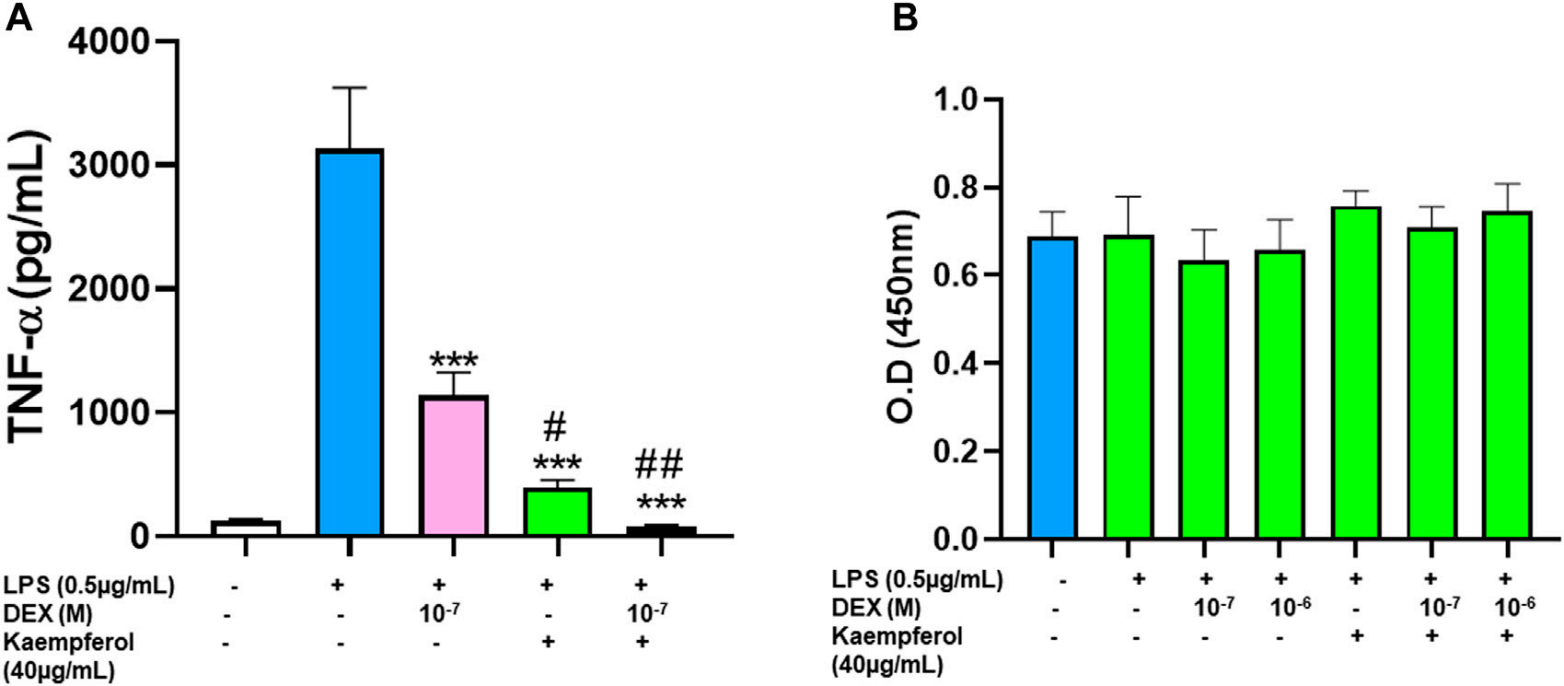
Effect of Kaempferol and dexamethasone co-culture on TNF- α production by RAW cells: Epimedium and dexamethasone synergistically suppressed TNF-α production RAW cells were cultured with Epimedium either alone or in combination with Dexamethasone for 24 h. The cells were stimulated with LPS (0.5 pg/m1). The supernatant were harvested and TNF-α levels were measured by ELISA. **(A)** Dexamethasone (10^–7^,10^–6^ M) and Kaempferol (40 pg/m1) significantly reduced TNF-α levels. When Kaempferol and dexamethasone was added together, the TNF-α levels were suppressed by 97%. **(B)** Cell viability with CCK8 assay showed no cell cytotoxicity. Data represents triplicate experiments and expressed as mean ± SD. "'*p* < 0.001 *vs.* control (without LPS), "*p* < 0.05; "*p* < 0.01 *vs*. Dex (10^−7^ M).

Based on kaempferol’s pharmacological profile, we then tested the effects of combining kaempferol with dexamethasone on TNF-α production. Dexamethasone and kaempferol each showed significant suppression of TNF-α production, respectively compared with untreated cells (Figure 6A *p* < 0.001). Interestedly, TNF-α production was further reduced and approached baseline levels when dexamethasone and kaempferol were used in combination (*p* < 0.001 *vs*. dexamethasone alone, kaempferol alone treated cells). This inhibition did not cause any sign of cytotoxicity (Figure 6B).

### 3.8 Kaempferol inhibits CYP3A4 activity in a dose-dependent manner

**FIGURE 7:**
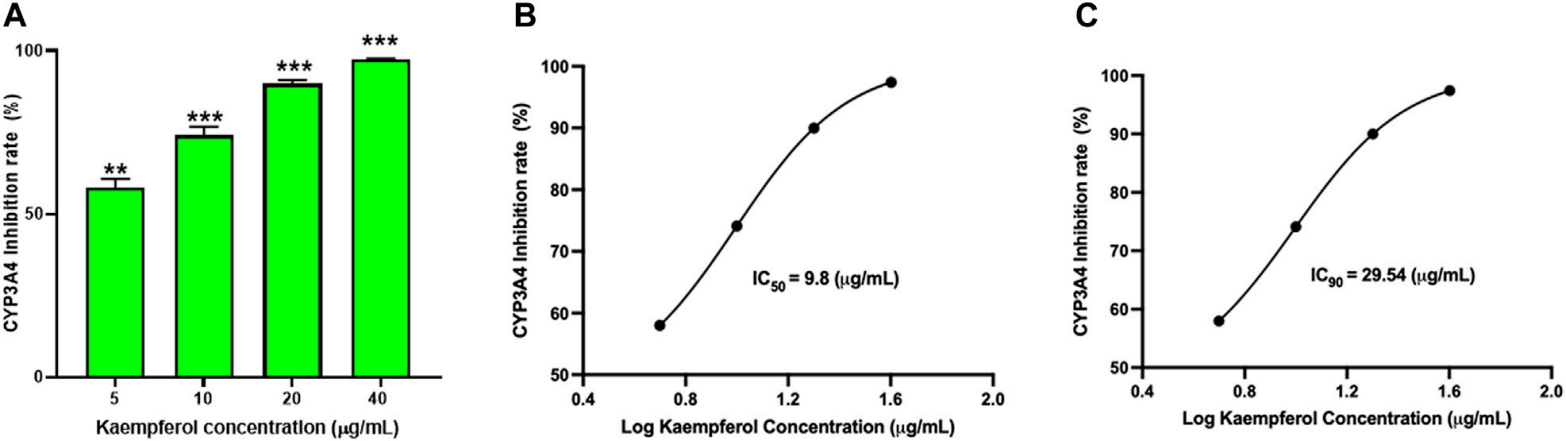
Kaempferol dose dependent inhibition of CYP3A4 activity: The inhibition of CYP3A4 was measured by commercially available Vivid assay kit as per manufacturers instruction. **(A)** Kaempferol showed dose-dependent suppression of CYP3A4 enzyme activity. The IC_50_ and IC_90_ were calculated to be 9.8 pg/mL and 29.54 pg/mL respectively **(B,C)**. Data represents triplicate experiments and expressed as mean ± SD. "*p* < 0.01; ***p < 0.001 *vs*. control.

To determine if kaempferol inhibited CYP3A4 activity, a Vivid CYP450 kit was used to measure CYP3A4 activity at different concentrations of kaempferol. Kaempferol dose-dependently suppressed CYP3A4 activity (Figure 7A, *p* < 0.01). The inhibition rate was 58.0%, 74.1%, 89.9%, and 97.4% at an incremental concentration of 5.0, 10.0, 20.0, 40.0 μg/mL respectively (Figure 7A). Based on the kaempferol inhibition of CYP3A4, we calculated a IC50 and IC90 of 9.8 μg/mL and 29.54 μg/mL, respectively (Figures 7B, C).

### 3.9 Computer docking of kaempferol and CYP3A4

**FIGURE 8:**
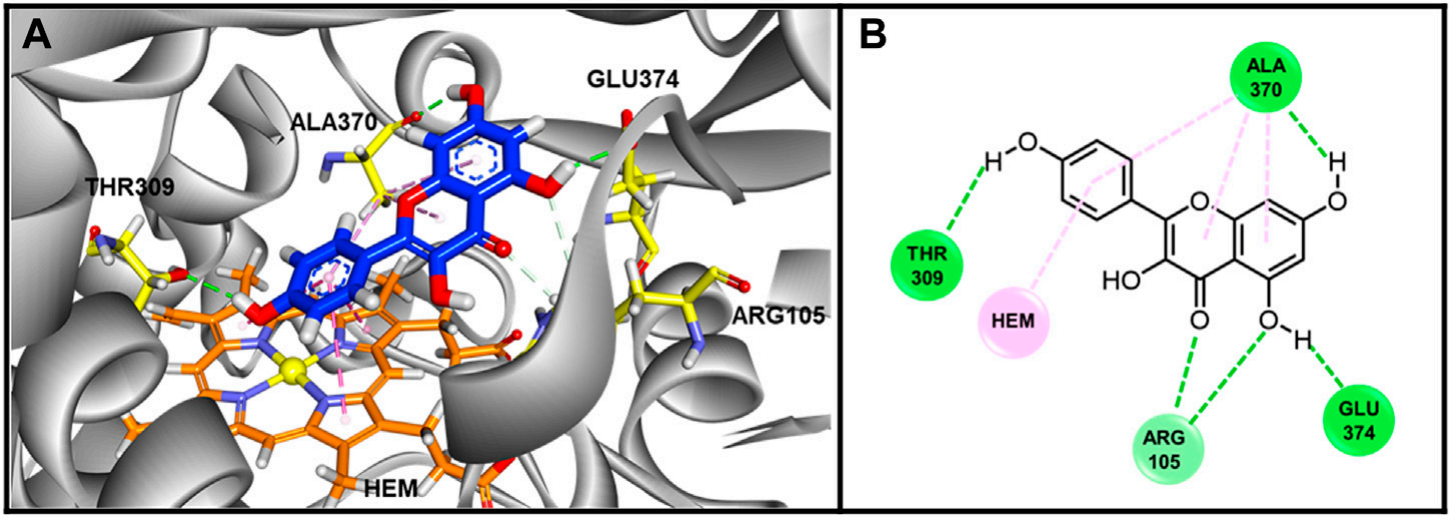
The Binding conformations of compound Kaempferol with enzyme CYP3A4. **(A)** 3D Binding pose of Kaempferol in inhibited binding domain of CYP450 3A4. For Kaempferol, carbon and oxygen are highlighted in blue and red, respectively. For amino acids of protein, carbon, oxygen and nitrogen are displayed by yellow, red and blue, respectively. For HEM molecule, the orange, blue and yellow color stand for carbon, nitrogen and iron ion. **(B)** Binding interactions between Kaempferol with residues of CYP450 3A4. The green and pink lines stand for hydrogen bonds and hydrophobic interactions. The docking score was −44.7332 kJ/md, showing good binding affinity between kaempferol and CYP3A4.

To further understand how kaempferol interacts with the CYP3A4 protein structure, we conducted *in silico* molecular docking/modeling. The docking score is −44.7332 kcal/mol, implying strong binding affinity between kaempferol with CYP3A4. The binding conformation was illustrated in Figure 8. Hydrogen bonds between kaempferol and THR390, ALA370, ARG105 and GLU 374 were formed, which stabilize the configuration of kaempferol. There is π-π stacking interaction between benzene group of kaempferol with HEM molecule in CYP450 3A4. The hydrophobic interactions were found between kaempferol with ALA370. The kaempferol completely occupied the catalytic sites of CYP450 3A4, preventing enzyme binding with its substrates, which likely explains part of the mechanism of how kaempferol influences CYP450 3A4.

## 4 Discussion

Corticosteroids are a mainstream anti-inflammatory class of drugs and CYP3A4 is the major drug metabolizing enzyme of corticosteroids. Studies have shown that dexamethsone paradoxically enhances CYP3A4 gene expression (Pascussi et al., 2001), consequently reducing the anti-inflammatory capacity of corticosteroids. Therefore, a natural product that enhances the anti-inflammatory capability of corticosteroids by modulation of CYP3A4 will be an important strategy to enhance the anti-inflammatory effects of corticosteroids. Previous studies have investigated the effects of natural products including drug interactions however there were no reports on potential therapeutic implications of these interactions (Mahgoub, 2002; Melillo de Magalhães et al., 2012; Huang et al., 2013). CYP3A4 is the dominant CYP3A family enzyme expressed in the human liver and gastrointestinal tract (Horsmans, 1997). Pharmacogenomic variations of CYP3A4 in human have been implicated in the metabolism of many drugs and in drug-drug interactions which could have important implications for the clinical use of drugs in combination therapy (Gurley et al., 2002; Delgoda and Westlake, 2004). *In vivo* studies have shown that Panax ginseng (Malati et al., 2012) and, Angelica (Ishihara et al., 2000) respectively induces and inhibits CYP 3A in the liver and gastro intestinal tract, therefore patients taking them in combination with other substrates that are metabolized by CYP3A must be closely monitored for adequate therapeutic response to the substrate medication. Therefore, due to the wide spectrum of substances processed by CYP3A4, interferences and interactions are not uncommon.

In this study, we demonstrated for the first time that epimedium dose-dependently inhibited the CYP3A4 enzymes with IC50 value of 95.73 μg/mL and an IC90 value of 216 μg/mL, respectively. Due to its inhibitory effect on the CYP3A4 enzyme, we hypothesized that epimedium would regulate CYP3A4 mRNA expression in the presence of dexamethasone. Therefore we measured the effect of epimedium alone and in combination with dexamethasone on the mRNA expression of CYP3A4 using an HepG2 cell line because it is a suitable surrogate of primary human hepatocytes to determine changes in CYP3A4 expression in the human body (Li et al., 2013). The results showed that epimedium inhibited CYP3A4 transcription without any cytotoxicity. Dexamethasone alone at a low concentration of 10 μM induced CYP3A4 mRNA, similar to rifampin (a CYP3A4 enhancer (Kobayashi et al., 2019)). However, in combination with epimedium, we observed 50% less suppression of CYP3A4 mRNA expression. This suggests that epimedium strongly inhibits CYP3A4 activity and would potentially influence the metabolism and increase the bioavailability of dexamethasone, providing a synergistic anti-inflammatory effect. To confirm this synergistic effect, we measured TNF- α production when epimedium is combined with dexamethasone. Thus, we tested the ability of epimedium to suppress LPS-induced TNF-α in macrophages. First, we showed that dexamethasone at 10^–7^ M caused a 20.4% inhibition of TNF-α and at a higher concentration of 10^–6^ M it caused a 63.2% inhibition of TNF-α. While epimedium at 125 μg/mL resulted in a 78.3% inhibition. In combining epimedium with dexamethasone at 10^–7^ M and at 10^–6^ M, we observed further suppression of TNF- α regardless of the difference in concentrations. Thus, our results confirmed the synergistic anti-inflammatory effect of epimedium in combination with dexamethasone. This implies that dexamethasone can be used at a lower dose with epimedium to achieve a more potent inhibition of TNF-α, which is clinically significant. The combination might serve to reduce the potential side effects of corticosteroids while maintaining or even enhancing its anti-inflammatory effects, this warrant further clinical investigation.

Furthermore, we used TCSMP database to screen bioactive compounds of epimedium. We identified 11 candidates based on the criteria of oral bioavailability and drug like index. We selected the commercially available compounds icariin, anhydroicaritin, kaempferol, and quercetin from the 11 compounds to measure their anti-inflammatory effect on IL-8. Out of the four compounds tested, only kaempferol significantly reduced the expression of IL-8 and did so in a dose dependent and non-toxic manner. In addition, kaempferol in combination with dexamethasone synergistically suppressed TNF-α production in macrophage cell line supporting that kaempferol is one of the epimedium active compounds. Furthermore, we showed that kaempferol inhibited CYP3A4 activity and in a dose dependent fashion with an IC50 of 9.8 mg/mL and IC90 of 29.54 mg/mL, which is approximately 10 X more potent than its parent extract epimedium. Using molecular docking we showed that kaempferol binds strongly to CYP3A4 by occupying the catalytic sites of CYP450 3A4, preventing enzyme binding with its substrates.

Human studies evaluating the bioavailability of kaempferol shows that the conjugated forms of kaempferol have a higher bioavailability than the free forms (Hollman et al., 1995; Hollman et al., 1996). Upon absorption, kaempferol is rapidly metabolized in the liver to form glucuronide methyl, and sulfate metabolites which are detected in blood and urine. Few studies that have investigated kaempferol have shown that the most available forms of kaempferol are–kaempferol glucoside and rutinoside in tea (Dabeek and Marra, 2019). Studies conducted by De Vries et al. (de Vries et al., 1998) examined the digestion and absorption of kaempferol from black tea in 15 participants. It was shown that urinary excretion of kaempferol was 2.5% of the amount ingested. The digestion and absorption of kaempferol were also assessed after an intake of 12.5 mg kaempferol from broccoli for 12 days, it was shown that the urinary excretion was 0.9%. There are limited clinical studies on the bioavailability of kaempferol and as such more studies are needed to investigate the bioavailability in a clinical setting. Our studies on kaempferol therefore provide an *in vitro* data of its effects on CYP3A4 and the results in this study will need to be further validated in a clinical setting.

Flavonoids are generally extensively metabolized by the liver (Mullen et al., 2006). There are few studies evaluating the bioavailability of kaempferol. In the study by (DuPont et al., 2004) the metabolites of kaempferol were assessed in plasma and urine after ingesting kaempferol-containing leafy vegetable endive. It was shown that kaempferol-3-glucuronide was the major metabolite of kaempferol found in both urine and plasma. 40% of total kaempferol was detected in plasma while 14% was detected in urine. This shows that kaempferol has the potential to reach inhibitory concentration. However, this area needs to be further explored. More studies need to be carried out to further understand the metabolism and bioavailability of kaempferol in the human body.

## 5 Conclusion

In conclusion, this study reveals, to the best of our knowledge for the first time, that epimedium suppresses CYP3A4 level, and thus enhance the dexamethasone effect on LPS-induced TNF-α production, probably through inhibition of CYP3A4 activity and upregulation of dexamethasone utilization. Our data illustrates the potential therapeutic value of epimedium in treating steroid-refractory diseases. Kaempferol is an effective candidate for the downregulation of IL-8 expression. Further studies using epimedium and kaempferol on HepG2 cell lines may provide relevant additional data on anti-inflammation, and *in vivo* study of dexamethasone in the presence of epimedium or kaempferol is warranted.

## Data Availability

The datasets presented in this study can be found in online repositories. The names of the repository/repositories and accession number(s) can be found in the article/Supplementary Material.

## Abbreviations

CS, Corticosteroids; CKZ, Chuan Ke Zhi; CYP450, Cytochrome P450; CYP3A4, Cytochrome P450 3A4; DL, Drug-likeness; DMSO, Dimethyl sulfoxide; IL-1β, Interleukin-1β; IL-8, Interleukin-8; LPS, Lipopolysaccharide; OB, Oral bioavailability; TCMSP, Traditional Chinese Medicine Systems Pharmacology; TNF-α, Tumor Necrosis Factor.
