# Supplementary figures and images for: Cytochrome P450 3A4 suppression by epimedium and active compound kaempferol leads to synergistic anti-inflammatory effect with corticosteroid
Source: Front Pharmacol. 2023 Jan 30;13:1042756. doi: 10.3389/fphar.2022.1042756 (PMC9922998; doi:10.3389/fphar.2022.1042756)

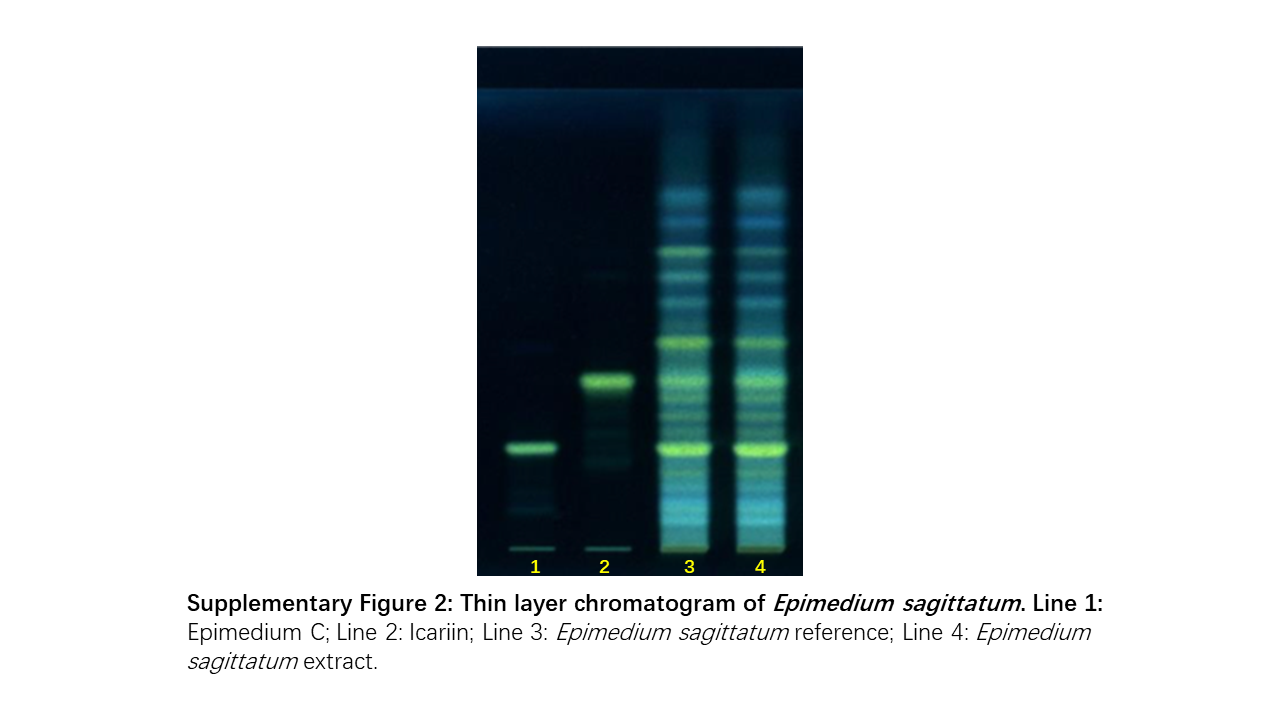

Supplement: Supplementary file 1 [file Image3.tif]

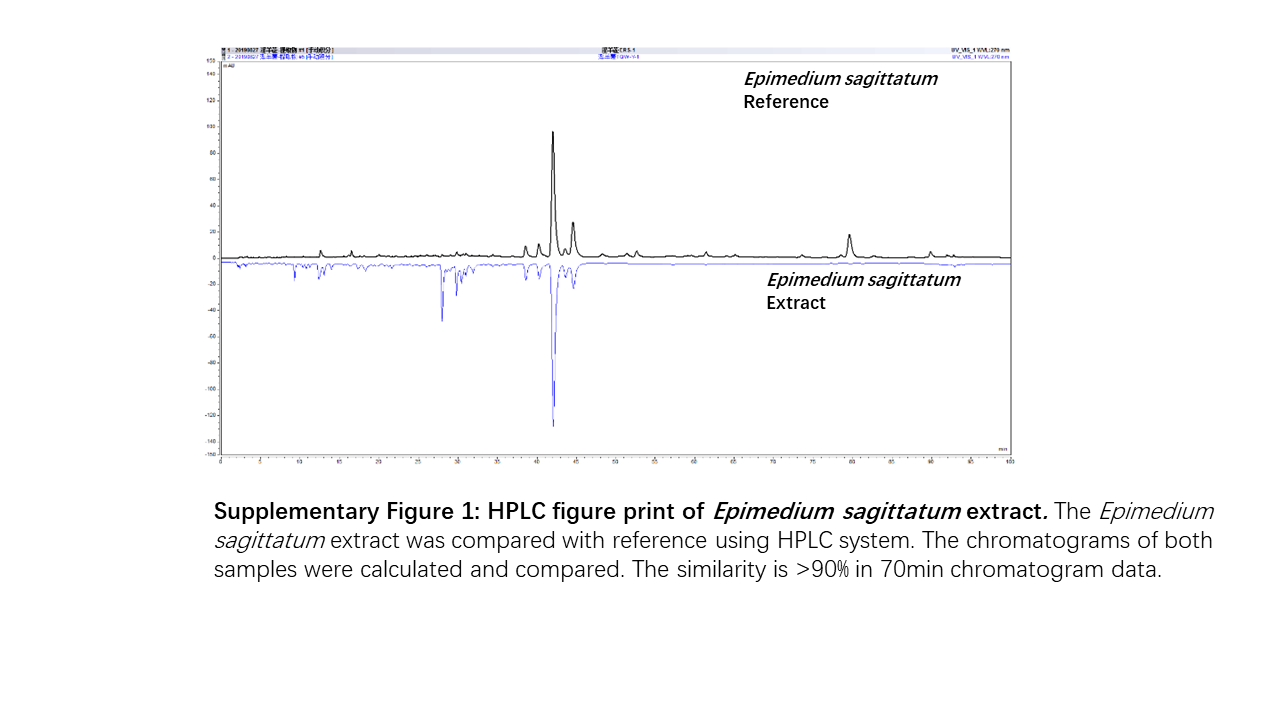

Supplement: Supplementary file 2 [file Image2.tif]

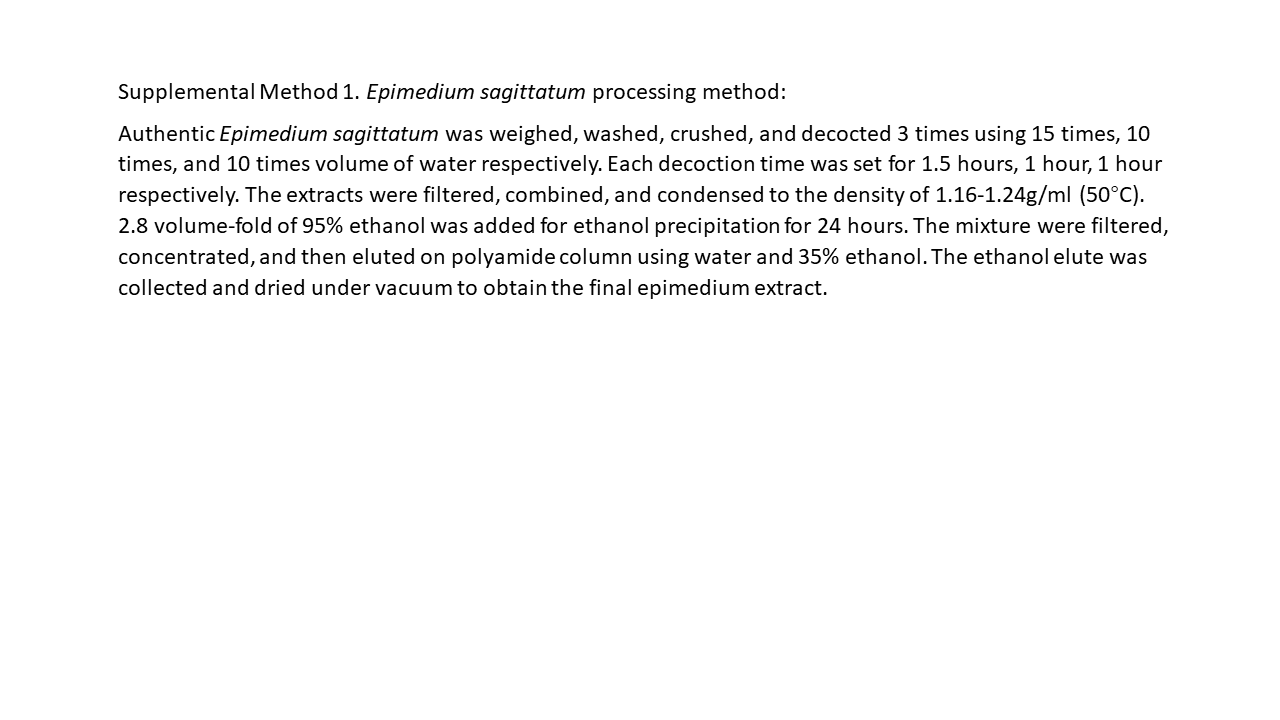

Supplement: Supplementary file 3 [file Image1.tif]
